# Semantic Registration and Discovery System of Subsystems and Services within an Interoperable Coordination Platform in Smart Cities

**DOI:** 10.3390/s16070955

**Published:** 2016-06-24

**Authors:** Gregorio Rubio, José Fernán Martínez, David Gómez, Xin Li

**Affiliations:** Centro de Investigación en Tecnologías Software y Sistemas Multimedia para la Sostenibilidad (CITSEM), Universidad Politécnica de Madrid (UPM), Edificio La Arboleda, Campus Sur UPM. Ctra. Valencia, Km 7, 28031 Madrid, Spain; jf.martinez@upm.es (J.F.M.); david.gomezs@upm.es (D.G.); xin.li@upm.es (X.L.)

**Keywords:** subsystem registry, subsystem discovery, service registry, service discovery, semantic interoperability, ontology, system of systems

## Abstract

Smart subsystems like traffic, Smart Homes, the Smart Grid, outdoor lighting, etc. are built in many urban areas, each with a set of services that are offered to citizens. These subsystems are managed by self-contained embedded systems. However, coordination and cooperation between them are scarce. An integration of these systems which truly represents a “system of systems” could introduce more benefits, such as allowing the development of new applications and collective optimization. The integration should allow maximum reusability of available services provided by entities (e.g., sensors or Wireless Sensor Networks). Thus, it is of major importance to facilitate the discovery and registration of available services and subsystems in an integrated way. Therefore, an ontology-based and automatic system for subsystem and service registration and discovery is presented. Using this proposed system, heterogeneous subsystems and services could be registered and discovered in a dynamic manner with additional semantic annotations. In this way, users are able to build customized applications across different subsystems by using available services. The proposed system has been fully implemented and a case study is presented to show the usefulness of the proposed method.

## 1. Introduction

A diversity of urban subsystems, such as Intelligent Transport Management systems [1,2], Smart Buildings systems [3], Smart Gird systems [4,5,6], Smart Outdoor Lighting systems [7] and Smart Home systems [8] are maturely developed in urban areas. Basically, each of them is managed by self-contained embedded systems and connected with Wireless Sensor and Actuator Networks (WSANs). Different smart subsystems can work effectively providing domain-specific services to citizens, but in an isolated manner. Unfortunately, collaborations and coordination between diverse smart subsystems are missing, even though they could potentially provide more citizen-friendly services by using data/services provided by different subsystems. This implies that cities are facing an unprecedented challenge, which is integrating fragmented smart subsystems and enabling cross-domain usages of services. A paradigm shift from conventional cities to “Smart Cities” has attracted a lot of interest from the research community, governments, and industry. This is an ongoing change that has been undertaken in many cities. For instance, in Spain, there are 65 cities integrated in RECI [9] which is the Spanish Network of Smart Cities. To turn conventional cities into smart ones, a system-thinking approach is needed to facilitate interconnections between different subsystems. A new platform aiming to connect different subsystems and represent a true “system of systems” integration has been developed in the Adaptive Cooperative Control in Urban (sub)Systems (ACCUS) project [10]. This platform, called Integration and Coordination Platform (ICP), emphasizes how to integrate different subsystems and enables the development of new applications across subsystems without interfering individual updates and internal policies.

The ICP is capable of providing a variety of functionalities in order to optimize combined performance of different subsystems, thus achieving more flexible, more efficient and more robust integrated urban systems and managing different emergent behaviors. The platform is conceived as a distributed and layered architecture which offers three main groups of functions:

- Core functions: they are referred to generic platform functionalities which are offered by the Runtime Environment of the ICP. Core functions are provided by the following software components: applications servers, service broker, service repository, service bus, data broker and management, policy management, security management, workflow engine, security management and message broker and database.
- Extension functions: they are referred to ICP specific functionalities that can be used by other services and applications. Extension functions are offered by info broker, control broker, subsystem monitoring, subsystems adaptors, ontology connector, programming API and ICP ontology.
- City configurations functions: these are city specific generic functionalities available for other ICP services and applications. They are provided by event detection, location detection, data analytics, situation awareness, and reasoning.

To ensure that the ICP could fulfill the aforementioned capabilities, a key premise is to address the heterogeneity (e.g., data formats and protocols) inherent to subsystems and services provided by different subsystems and provide a unified interface of reference for available subsystems and services within the ICP. With this reference, maximum reusability of available services provided by entities (e.g., sensors, or Wireless Sensor Networks) could be enabled so as to develop new cross-domain applications. Thus, an ontology-based and automatic system for registering and discovering subsystems and services is presented in this paper. This proposed system can be embedded into the ICP in order to discover and register available heterogeneous services and subsystems in a dynamic manner. The proposed system for subsystems and services registration and discovery is able to adapt to any change that occurs in subsystems and services. Additionally, in order to abstract the heterogeneity of subsystems and services and provide a common understanding to the ICP, this proposed system employs an ontological approach to provide a formalized model, named *new Subsystem and Service Oriented Ontology* (*nSSOO*), for the registry and discovery process. Thus, different subsystems and services could be semantically annotated and understood by the ICP and users as well. The proposed system for subsystem and service registration and discovery is playing an important role in ICP to enable the creation of real-time collaborative applications across subsystems by employing services available in the city. The proposed system is completely implemented and validated by using a traffic light control application use case.

This paper is organized as follows: Section 2 presents related work on existing platforms for Smart Cities and their solutions to register and discover subsystems and services within them. The proposed system for registry and discovery of subsystems and services within the ICP is shown in Section 3. Specifically, Section 3.1 highlights the main contributions that are provided by this paper. Section 3.2 shows system integration regarding Semantic Interoperability right afterwards. The newly proposed nSSOO ontology used to model the registry and discovery information base is introduced in Section 3.3. Section 3.4 provides a holistic view of the system and elaborates the specific software components that are involved. Workflow for the subsystem and service discovery and registration procedures is detailed in Section 3.5. Section 4 presents a use case about the traffic light control to validate the proposed system. Finally, in Section 5, conclusions are given and future work is pointed out as well.

## 2. Related Work

Technical interoperability levels have progressed in the last years with already mature solutions. However, semantic interoperability [11] remains as a key obstacle to the seamless exchange of data between services, applications or systems. However, some European projects are making significant progress in the development of platforms of interconnection, using Service Oriented Architecture (SOA) and semantic technologies to get semantic interoperability as a key factor of seamless interconnection and interoperability.

European FP7 projects that have worked on developing SOA-based platforms are as follows:

SOA4ALL [12] proposed a framework and software infrastructure that aims at integrating SOA and four complementary and evolutionary technical advances (Web, context-aware technologies, Web 2.0 and Semantic Web) into a coherent and domain-independent worldwide service delivery platform. Also, some semantic source components have been developed and the concept of “linked services” which is Semantic Web services building on the success of the Linked Open Data initiative. So, they are services that can consume Resource Description Framework (RDF) from the Web of Data and feed-back RDF to Web of Data. However, “linked services” is not the same concept as the services with semantic interoperability, especially if there are services working together that have different ontologies or no semantic representation at all.

TaToo [13] proposed a framework to allow third parties to discover environmental resources data and services on the web and add valuable information in the form of semantic annotations to these resources. This annotation allows improving the reasoning and inference power of the ontologies to create richer resource annotations. This process has the goal of optimizing discovery process of services, but not improving interoperability.

Cloud4SOA [14] proposed a solution based on the concept of using cloud computing under the paradigm of PaaS [15] to resolve the interoperability and portability issues that exist in current cloud infrastructures using the same technological providing a user-centric approach for applications that are built upon and deployed by means of Cloud resources.

SemanticHealthNet [16] proposed a set of resources to support semantic interoperability process for clinical and biomedical knowledge, but it cannot be considered as a platform.

LifeWear [17] proposed a middleware platform to interconnect wearable devices and sensors of a WSN using services semantically annotated in a compliant way to an ad-hoc ontology. The system shown in this manuscript is an important evolution of the results of this project.

Not only European projects have proposed platforms; Ryu proposed in [18] an Integrated Semantic Service Platform (ISSP) with IoT-based service support in a Smart City, addressing ontological models in various domains of a Smart City.

Hussian et al. [19] presented an integrated platform to be deployed in a Smart City. This platform could enable a unified and and people-centric access to all services provided by the Smart City. However, this proposed platform emphasized the integration of various healthcare systems within the Smart Cities. Thus, it lacks generality and it is not applicable while attempting to integrate a diversity of smart subsystems beyond healthcare systems.

A distributed platform called “Kalimucho” [20] was built to enable the design of context-aware applications based on heterogeneous devices in Smart Cities. This platform is ambitious in the sense that provides a tight collaboration between different subsystems, such as transportation and logistics, healthcare, and smart environments. The platform is conceived as “everything-as-a-service” which regards subsystems and services provided by corresponding subsystems as independent services. However, all those services are pre-registered in the platform. Dynamicity of registering and discovering new services is missing; also, semantic annotation for the different services is not considered in this proposal.

Furthermore, there have been efforts related to integration, in different ways, using semantic technics WSAN and services, as antecessors to the whole platform: Rodriguez-Molina et al. [21] proposed a semantic middleware for Wireless Sensor Networks, in order to provide integration of sensors in a body area network with other WSAN present in a smart city. Bispo et al. [22] proposed a more advanced model in Semantic Infrastructure for Wireless Sensors Networks (SITRUS) with semantic information processing to generate a semantic database focused on determining the reconfiguration of a WSAN combined with a message-oriented communication service and another one used for reconfiguration. Camarhina-Matos et al. [23] proposed using the concept of collaborative network for the integration of networks or WSAN belonging to different organizations, that involves mutual engagement of participants to solve a problem together, thus implying mutual trust and taking time, effort, and dedication. In this proposals, the network of each organization can be considered a subsystem and each application a service. Last years, with the rise of Software Defined Networks (SDNs) [24], there have been efforts bent on enhancing interoperability among the various heterogeneous wireless networks when control and information levels are separated. Kosmides [25] showed a system with a centralized network controller based in SDN applied to social networks as case of study, where the Smart City was divided in geographical zones, and each zone was considered as a subsystem. However, the concept of semantic registration and discovery of subsystems and services is not embedded in the development works implemented using SDN or Collaborative Networks.

## 3. Proposed System for Registration and Discovery of Subsystems and Services within ICP

In this section, a new proposal for a system made for semantic registration and discovery of subsystems and services within ICP is presented. Specifically, the main contributions that are provided by this paper are listed in Section 3.1. The integration system offering semantic interoperability is shown in Section 3.2. Section 3.3 is devoted to introducing the newly proposed nSSOO ontology. The system architecture of subsystems and service registration and discovery are described in Section 3.4. Finally, the specific procedures to facilitate the registration and discovery of subsystems and services are elaborated in Section 3.5.

### 3.1. Innovations

Some works shown in the previous section use registration systems developed ad-hoc or integrated in Application Servers as WildFly [26] or WSO2 [27], that they have their own registration service. However, to satisfy the registration and discovery system that ACCUS ICP needs, it is necessary to add three innovations.

The first innovation of the proposed semantic subsystem and service registration and discovery system is that it contributes to the integration and coordination of urban systems, connected to the ACCUS ICP, to build applications like monitoring, management and control that can reach beyond the borders of the individual subsystems and services. The proposed system contributes to cross-domain and cross-layer cooperation of urban subsystems and services by addressing different interoperability aspects, such as semantic interoperability.

Semantic interoperability provides means for seamlessly integrating urban subsystems, composing more complex functionalities from already existing subsystems and deploying converged scenarios. It will also enable the integration and deployment of present and future urban subsystems and processes in urban environments with little involvement from the side of either the developers or operators, in an automated way, based on common agreed ontologies and semantic artefacts.

Another aspect of interoperability addressed by the proposed system is the information and knowledge discovery. It enables every subsystem, service or application connected to the ACCUS ICP, to discover registered subsystems, services or applications, and obtain information about them, by sending a query request to the ACCUS ICP. It will respond, after giving authorization to the subsystem, with the information requested according to the defined subsystems and services ontology.

Information and knowledge discovery enables, in turn, the development of applications that combine the information about services and subsystems provided by the ACCUS ICP with the purpose of offering more complex services able to provide functionalities that subsystems and services cannot provide separately, and facilitating, thus, service composition.

The second innovation of the proposed semantic subsystem and service registry and discovery procedure, is its capability of enabling a distributed control, management and optimization infrastructure, along with the algorithms and tools required to create highly advanced urban control functions, which will be implemented through the introduction of cooperation extension over multiple urban subsystems, system layers and domains. This will improve the performance of combined urban systems at run time.

The third innovation of the proposed system is that it ensures the development and application of methodologies and tools for the implementation of real-time collaborative applications for system of systems. The methodology and tool innovation covers the entire life-cycle (i.e., from design to operation, maintenance and possibly retrofitting) of the applications developed for the integrated urban subsystems domain.

### 3.2. Seamless Interconnection and Semantic Interoperability

In system of systems, interoperability is the ability of two or more subsystems or components to exchange information and to use what has been interchanged [28]. According to the features shown in this paper about the ICP, the most accurate model is the Level of Conceptual Interoperability Model (LCIM) [29] because it provides a framework that divides interoperability problems into different levels; at each level, interoperability problems can be settled and a solution can be developed to solve interoperability problems that belong to that level. It contains seven levels: Level 0—No interoperability; Level 1—Technical interoperability: networks and standard communication protocols enable the interchange of data between systems; Level 2—Syntactic interoperability: adds a common structure and data format to the data interchanged in an unambiguous way; Level 3—Semantic Interoperability: adds a common interpretation of data interchanged, the meaning of information exchanged between systems is defined in an unambiguous way; Level 4—Pragmatic Interoperability: systems are aware of the specific use of exchanged data by other systems; Level 5—Dynamic Interoperability: systems are able to understand the change of states in each element depending on the decisions taken according to the use of data; Level 6—Conceptual Interoperability: global interoperability.

The system of registration and discovery proposed in this paper allows the ICP to solve technical, syntactic and semantic interoperability issues. When the meaning of data is shared among services the content of the information exchange among them is unambiguously defined, so common interpretation of the data is guaranteed. ICP uses data from several different data sources, various sensors are connected to these data sources and each sensor uses different data format, which is further processed. Various types of data are used, creating a system able to combine these data. With the data integration system it is possible to connect several heterogeneous systems and create one large system. Getting is relatively simple when the integration platform is created before the services. However, when subsystems and services are already installed and were designed without knowledge that in the future they would be integrated in an ICP merging all of them in a single platform can be a challenging task.

Another important achievement of semantic interoperability made through ICP is achieving semantic interoperability without the need to change anything regarding the mode of operation, communication, data management subsystems and services already installed in the city.

As data sources are highly heterogeneous, the ICP uses a Common Data Model as a common layer to interchange information among data sources (namely, services and subsystems).

The Common Data Model uses a shared ontology with adaptation of the information provided by subsystems and services, as each of them can provide the information in their own ontology or even in their own format, which might be non-compliant with any ontology.

**Figure 1:**
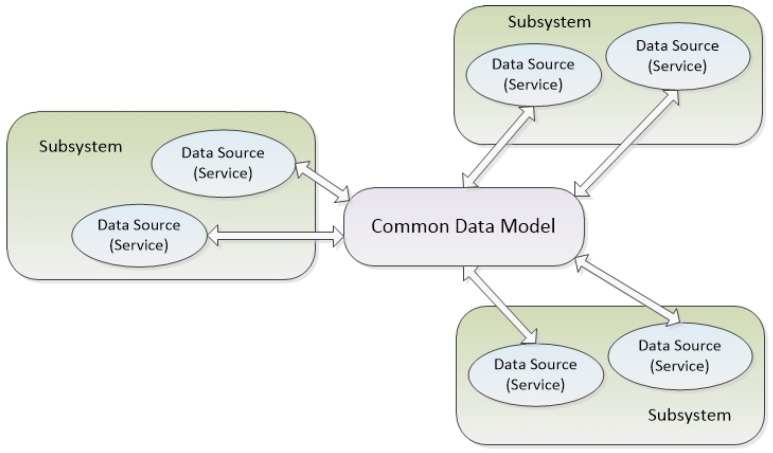
Common data model.

**Figure 2:**
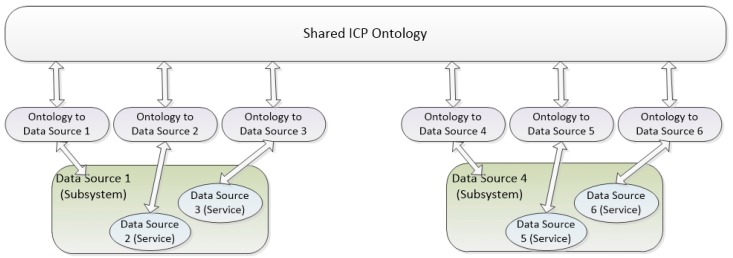
Integration system regarding Semantic Interoperability.

Using the schemas depicted in Figure 1 and Figure 2, all subsystems and services can be registered, discovered and used with the semantic capabilities established in the shared ICP Ontology. In this way, semantic interoperability is guaranteed, but with the advantage of not requiring any of the systems and services included in the smart city to modify their own syntax.

This shared ICP Ontology has a part specifically developed to register and discover subsystems and services in the ICP. It is specified in the next section.

### 3.3. Ontology Specifications

When attempting to provide an interoperable and formalized knowledge model for subsystem, service registration and discovery processes, the new Subsystem and Service Oriented Ontology (nSSOO) is proposed. The nSSOO is conceived to comprehensively and semantically describe a variety of features about subsystems and services owned by the complex urban system. This proposed ontology makes possible the integration and registration of information provided by sensors or subsystems. Originally thought for services offered by low capability devices (sensors, PDAs, RFID tags, etc.), this ontology can also be applied to services based on normal devices or subsystems (Smart Home subsystem, Smart Traffic subsystem, etc.), as in the ACCUS project.

**Figure 3:**
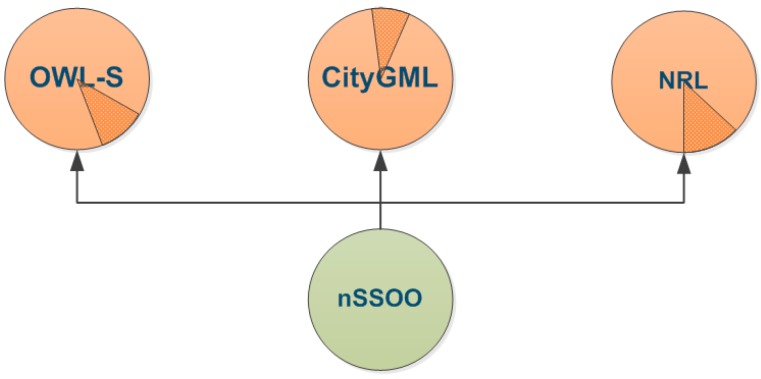
Proposal of nSSOO.

Figure 3 shows the hierarchical composition of the proposed nSSOO. Generally speaking, a few concepts of nSSOO are inherited from three existing and widely used ontologies which are Semantic Markup for Web Services (OWL-S) [30], City Geography Markup Language (CityGML) [31] and Security Ontology for Annotating Resources (NRL) [32]. The reusability of OWL-S, CityGML and NRL reduces the workload of developing the nSSOO and further expands its interoperability to a higher level.

The software tools used here are as follows:

- OWL-S. This ontology is used to describe semantic web services. It enables users and software agents to automatically discover, invoke and compose web resources while offering services.
- CityGML. This ontology models 3D cities taking into account multiple features, such as city geometry, topology, semantic features, and appearance characteristics. The ultimate aim of the development of CityGML is providing a common understanding for the basic entities, attributes, and relations of a 3D city model.
- NRL. It describes different types of security information including mechanisms, protocols, objectives, algorithms and credentials in various levels of detail and specificity. NRL is comprehensive, well-organized and expressive enough to describe security policies.

**Figure 4:**
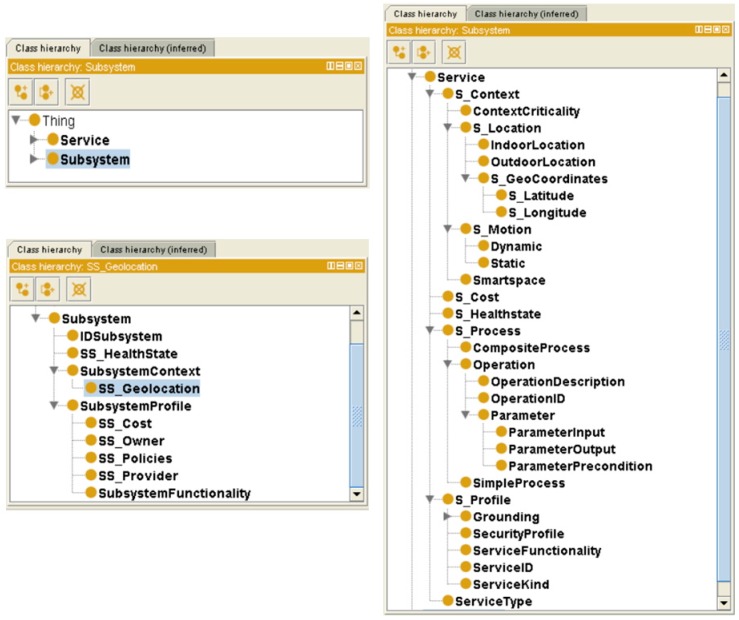
The visualized overall structure of nSSOO.

The most coarse-grained concepts are *Subsystem* and *Service*, which form the entire nSSOO ontology. In the following, the top-level concepts of *Subsystem* and *Service*, as shown in Figure 4, are broken down and their associated subclasses are explained in detail, along with descriptions for relationships/object properties which reflect the connections between them with the aim of providing a better understanding of the whole proposal. The primary principle of designing the nSSOO ontology is assigning different concepts with intuitive terms so that their meanings and intentions can be easily revealed. To make a clear distinction between service- and subsystem-owned ontology elements, prefixes “S_” and “SS_”, as abbreviations of service and subsystem, are attached to the corresponding ontology elements.

#### 3.3.1. Subsystem-Related Ontology Part

The concept of *Subsystem* represents the collection of city-owned subsystems that makes measurements and provides data about specific domains (e.g., weather subsystem, smart home subsystem, intelligent transport system etc.).

**Figure 5:**
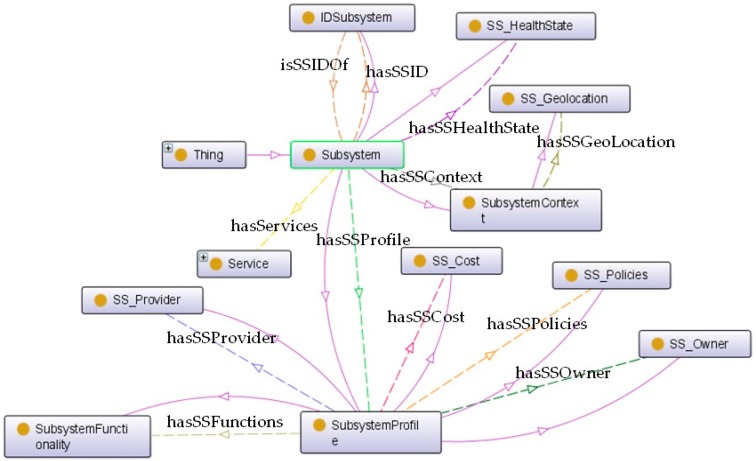
The internal structure of Subsystem.

*Subsystem* class can be unfolded into four main subclasses (see Figure 5, where the internal composition of *Subsystem* is presented with intuitive names for subclasses and relationships):

- *SubsystemContext*: the conditions in which the subsystem is provided. It is linked with *Subsystem* by an object property named *hasSSContext*.
- *SubsystemProfile*: descriptive information about the subsystem such as functionality, cost, provider, owner or usage policies. The *Subsystem* is interrelated with this class by using a *hasSSProfile* relationship. It is worth mentioning that the concepts of *SS_Geolocation* and *SS_Policies* are extracted from CityGML and NRL, respectively.
- *SS_HealthState*: information about the current health state of the subsystem. This class is connected with *Subsystem* via a *hasSSHealthState* relationship. Four different states (as potential individuals of *SS_HealthState*) are defined to describe the real status of a subsystem: *Installed* (it implies the subsystem is installed and ready to start once it receives an authorized command), *Active* (it states the subsystem is effectively running), *Suspended* (if subsystems are not required to run continuously, it is possible to make requests to pause them at any time during the active state) and *Stopped* (all the operations are stopped).
- *IDSubsystem*: a unique identification number to distinguish the subsystem. A pair of inversive (*owl:InversiveOf*) relationships (namely, *hasSSID* and *isSSIDOf*) dynamically links *Subsystem* with *IDSubsystem*.

#### 3.3.2. Service-Related Ontology Part

**Figure 6:**
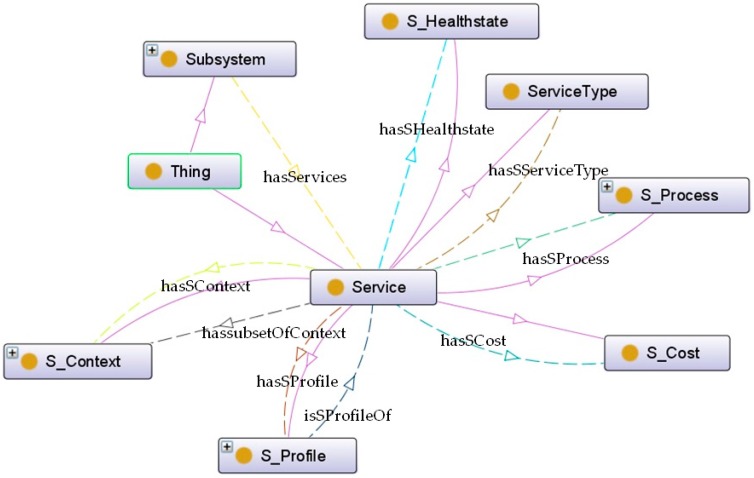
Top Level hierarchy of Service.

The concept of *Service* denotes all kinds of services available within the urban system, either provided by subsystems or the ACCUS ICP platform. *Service* can be classified into six major categorizations which are *S_Cost*, *S_Context*, *S_Process*, *ServiceType*, *S_HealthState*, and *S_Profile*, as shown in Figure 6. Each subclass describes the feature of *Service* from a different point of view so that the definition of *Service* can be comprehensively represented in this model. In the following section, the breakdown of each classification will be presented.

**Figure 7:**
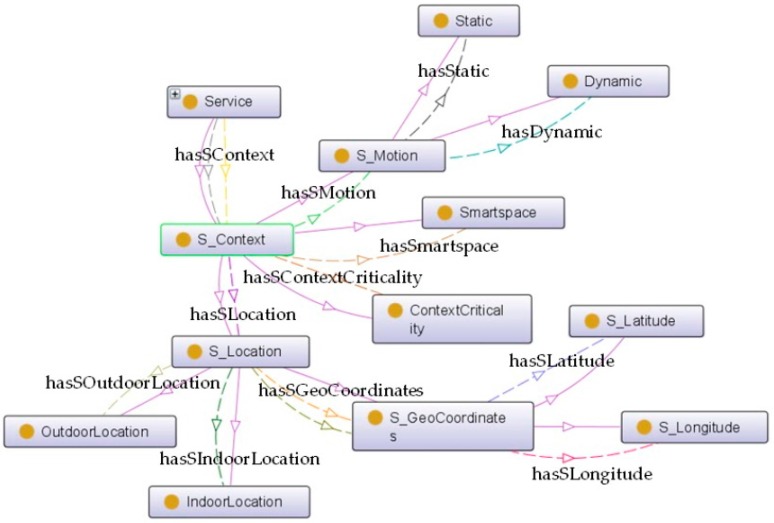
Internal structure of S_Context.

**Figure 8:**
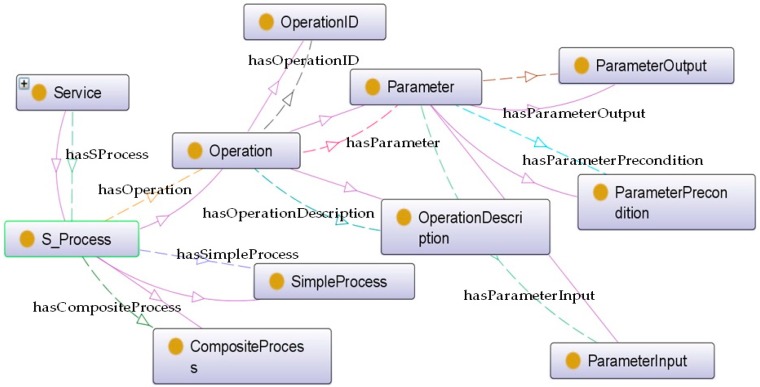
Internal structure of S_Process.

**Figure 9:**
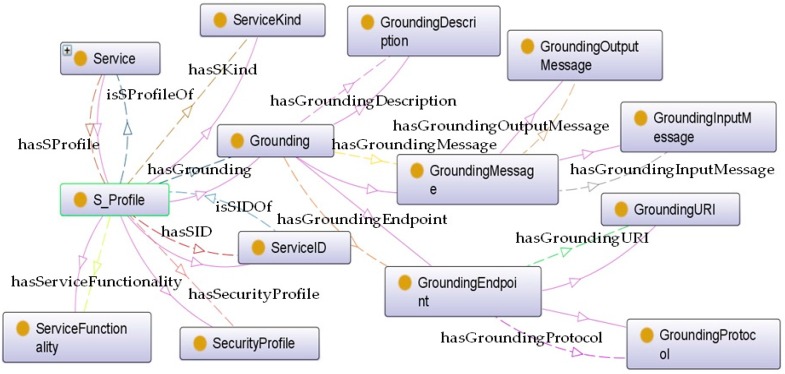
Internal structure of S_Profile.

The elements that make the top level hierarchy of service are:

- *S_Cost.* It is interrelating with *Service* via a *hasSCost* relationship; this class indicates the fee to be charged to users for using the service.
- *S_Context. Service* is connected with this class by a *hasSContext* object property. It expresses the environmental conditions involved to provide the service. More details can be visualized in Figure 7. For instance, if the service is *Static*, its functionality is always provided in the same location. Otherwise, if it is *Dynamic*, such as in the case of services provided by wearable devices where the location can change, it also contains information about the *ContextCriticality* declaring whether the context is critical for the service operation or not. The *S_Location* of the service depicts whether it is provided at an indoor or an outdoor location (split into *IndoorLocation* and *OutdoorLocation* classes respectively). The *S_Geocoordinates* of the service and *Smartspace* are able to provide a unique identifier of the service context.
- *S_Process*. The element *Service* is connected with this class that provides a complete description for the logic of *Service*, via a *hasSProcess* relationship. More details can be visualized in Figure 8. The *S_Process* class is refined into atomic and aggregated/complex processes. An atomic process (*SimpleProcess*) directly takes the information generated by the environment and executes the appropriated treatment to provide the functionality. On the contrary, the aggregated process (*CompositeProcess*) provides the new functionality by composing several atomic processes. Besides, the term *Operation* makes additional descriptions for service operations. More specifically, it provides a description of the methods the service provides (*OperationDescription*), an ID for each operation (*OperationID*), and information about used parameters including input and output parameters (*ParameterInput* and *ParameterOutput*, respectively) as well as the parameter preconditions (*ParameterPrecondition*).
- *ServiceType*. This concept aims to specify the concrete type of service. This classification considers service from its source, either provided by subsystems or by the ACCUS ICP. The connection between *Service* and *ServiceType* is established by an object property named *hasSServiceType*.
- *S_HealthState*. Similar as *SS_HealthState*, the class of *S_HealthState*, linking with *Service* by a *hasSHealthsate* relationship, describes the current state of *Service*.
- *S_Profile. Service* is interrelated with the *S_Profile* class via *hasSProfile* and *isProfileOf* relationships. Different features of the service are described and attributed in *S_Profile*. As shown in Figure 9, *S_Profile* states the *ServiceID* (a unique identifier for distinguishing the service), the ***ServiceKind*** (a more detailed specification for the type of service which differentiates it from the ontology's point of view, being either ACCUS-compliant or non-compliant, having the service using another ontology or not), the *ServiceFunctionality* (description of what the service is capable of doing), the *SecurityProfile* (description of the security features under which the service will be provided; this concept can be further extended by NRL), and *Grounding* (particular protocols used between the service and service consumers). Regarding the *Grounding* concept, it contains a more specific description (*GroundingDescription*) of the protocol, the URI (***GroundingURI***) and the protocol (*GroundingProtocol*) of the endpoint where the application is running and also the input (*GroundingInputMessage*) and output (*GroundingOutputMessage*) messages exchanged between the service and service consumers (see Figure 9).

### 3.4. Architecture of the Proposed System and Component Specifications

The ICP must provide a set of functionalities so that all subsystems belonging to a city will be properly operated. Also, it should support the development and deployment of (cross-domain) Smart City applications in any urban environment to enable the user to generate new services and applications which, in turn, will be integrated in the ICP. To achieve this, it is necessary to execute the next sequence of actions: (1) identification of ACCUS subsystems, services and applications; (2) identification and availability of external systems; (3) identification and availability of required infrastructure; (4) identification of information interchanged between ACCUS ICP and subsystems and services and (5) information provided by ACCUS ICP to cross-domain applications.

Taking into account the previously mentioned characteristics and the analysis of the information of different subsystems and services in the city, six technical features have been considered in an ICP: (1) Information and interaction, since ICP must provide services (annotated in an ICP ontology-compliant way) to enable the interaction among applications, applications and subsystems, and finally among subsystems if necessary. Two interfaces support that interaction: (a) Interface Applications—ICP, used to identify all requirements associated with the interaction among applications and ICP, considering that the information exchanged among them must be compatible with the ACCUS ontology with the idea of guaranteeing interoperability and an easy and seamless connection/disconnection of applications to the ICP. The features related with these requirements are communication with applications, an API provided to the applications and the relation with City State Database, CSDB, that stores all measurements and configuration of all WSANs and all sensors available in all subsystems deployed upon the Smart City; (b) Interface ICP-Subsystems, used to identify all requirements associated with the interaction among ACCUS ICP and subsystems; this interaction includes getting and sending information from/to the subsystem, as well as managing and controlling it, taking into account that not all subsystems are able to use the ACCUS ontology. Both interfaces must be used in the registration and discovery system, since all services and subsystems are registered and discovered by ICP while, on the other hand the cross domain application generated by users discovers the information of the services throught the semantic register and must be registered in this system, to let it be discovered by other cross domain applications; (2) Adaptive control, as the running and operating circumstances of services, subsystems applications and the own platform may vary over time, is also born in mind. The ICP has to adapt its operation to such changes; also, the control of all aspects related with the subsystems and services involved in the city is a key characteristic. Therefore, the control of the whole city depends on the ICP. What is more; (3) Security and Safety are a major concern, since the ICP is exposed to many security threats due to the security breaches likely to appear because of its dynamic and heterogeneous nature, as well as the fact that it is going to be usually operated by non-professional users in security issues; (4) Management to provide integrated management capabilities has also been conceived, as it allows both the platform as a whole and each of its software components to be managed. Applications, services and subsystems that make up the platform to suit the city are in permanent evolution too, so all the software updates and the connection/disconnection of functionalities should be done with a minimal impact on the normal operation of the platform when a new version is updated. In any case, it must be possible to return to a stable version, should significant problems appear in some updated components; (5) Development is eased by a virtual environment provided to simulate the real behaviour of the ICP. In this way, users can test the functionality of their new services or applications in a simulation environment before moving them to the operational phase; (6) Dependability [33] is employed to establish availability, reliability, safety, integrity and maintainability as its key aspects. In order to achieve this, the ICP provides self-monitoring, application state replication, plug-and-play and dynamic resource assignment.

As ICP should interconnect different city-owned subsystems where many services and applications are available but use different data formats, ICP has to create “a concept of system” which is able to combine all data formats to provide only a generic interface to applications. Therefore, the ICP must provide LCIM 3 (semantic interoperability), thus offering knowledge inference from heterogeneous data, storage of semantically enhanced data of legacy services and subsystems, integration of data and usage of data in cross-domain applications.

**Figure 10:**
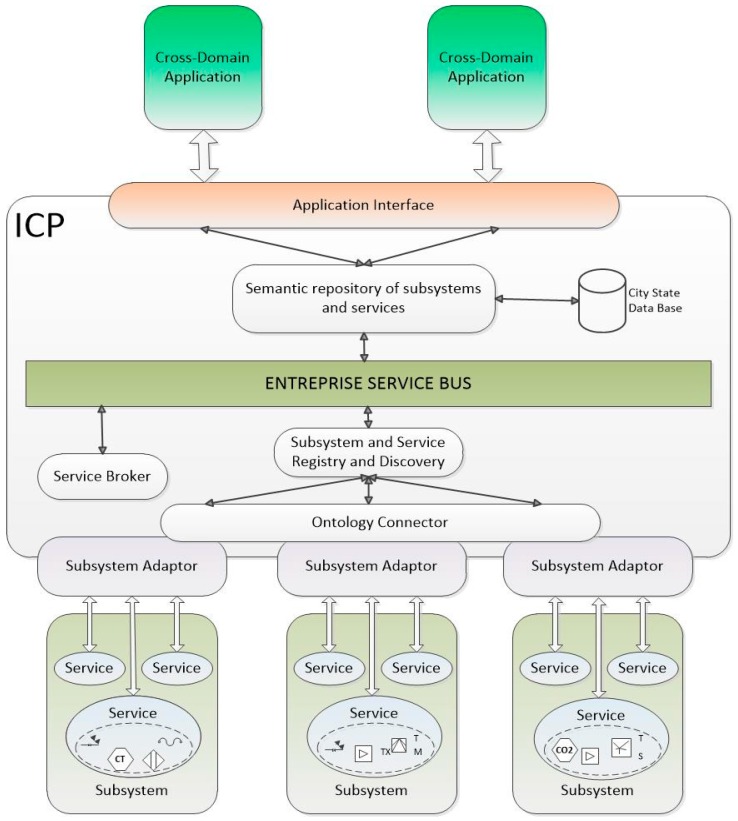
Proposed Integration and Coordination Platform regarding Semantic Interoperability.

In order to provide semantic interoperability it is proposed that the ICP applies a global ontology, showed in Figure 10. In terms of semantic interoperability, the ontology defines the vocabulary to exchange queries and assertions among applications. Ontological commitments are agreements to use the shared vocabulary in a coherent and consistent manner [11]. Services and applications sharing a vocabulary do not need to share a knowledge base. It is not necessary to know all the characteristics of the remaining components.

If all the functions described above are to be performed, the first action to be taken is ensuring interoperability among all the Smart City systems and applications. It is strictly necessary, first of all, that the ICP itself has its own complete system of registration and discovery of subsystems and services, which it is proposed in this paper. Figure 10 shows the architecture of the whole system of ICP related to registry and discovery.

The first step to discover all subsystems connected to ICP and the services provided by each subsystem is taken by the component Subsystem and Service Registry and Discovery through Interface ICP—Subsystems. A key point is that this component works in real time. Later, each service or subsystem will provide their own description in a compliant way to nSSOO or not. Regardless of what is used, ICP provides semantic interoperability and seamless interconnection between applications and services embedded in subsystems. In order to perform semantic mapping for data interoperability enabling the transparent sharing of information among subsystems in the smart city, the mail requirement is the translation of the output of subsystems and services that are going to interchange information among them. The translations of this service are used for the registration and discovery of services and subsystems in the service repository. This fact can be achieved by parsing XML data to RDF. This way, semantic experts can define the XML to RDF mapping and non-semantic experts can work with XML files avoiding the effort of analysing ontologies for each subsystem. The service is provided by the components Subsystem adaptor and Ontology Connector. The first one facilitates the connection of subsystems and their services, and provides all necessary functions of adaptation and coordination. It is a very relevant component since it is in charge of the transformation of the information provided by services and subsystems to the ICP. The second one handles the translations of the data format which will be added to the Semantic Repository, when data are delivered in a known XML-like format [34]. These translations from XMLs to OWL or RDFs will be made using a mapping file which describes the transformation between the elements of source XML to an instance of the global ontology. A mapping file must be defined for each type of XML instance. When the description of the service or subsystem is available for the Subsystem and Service Registry and Discovery, it will establish a connection trough the Enterprise Service Bus with the component Semantic Subsystem and Service Repository to store the semantic description of services and subsystems registered in the ICP, according to the global ontology defined for the system. When a new subsystem or service is discovered by the Subsystem and Service Discovery component, its semantic description (profile) will be stored in this repository. Enterprise Service Bus provides interconnection and cooperation of the components based on a paradigm of message interchange; the ESB selected in ACCUS ICP is JBoss [35], which was preferred over two competitive alternatives: WSO2 and MULE [36]. Obviously, the component Service broker is necessary to orchestrate services and mediate between different software protocols if necessary.

Once all services and subsystems are registered, the Cross-domain Applications through the Interface Applications-ICP can execute SPARQL [37] queries and get their needed results in order to generate new applications or services, that, at the same time, will be registered.

This mode of operation allows the permanent update of all services, subsystems and applications of the Smart City.

### 3.5. Procedure Specifications: The Specific Workflow of Registering and Discovering Services and Subsystems

In this section, the specific process to register and discover subsystems and services is introduced.

#### 3.5.1. Subsystems Registration

**Figure 11:**
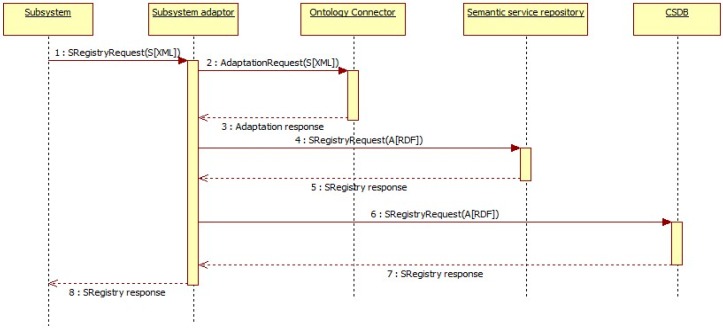
Subsystem registratiom sequence diagram.

Once all subsystems and ACCUS ICP are up, subsystems must start with the registration procedure one by one. As shown in Figure 11, first, each subsystem sends a registration request to the ACCUS ICP which includes the subsystem profile in an XML document. The subsystem profile contains information to identify itself, such as functionality, geolocation, health state, provider or input and output parameters. This information is not compliant with the ACCUS ontology and therefore, it must be adapted to the ACCUS ontology for the registration subsystem and stored in the CSDB. This adaptation is done using the ontology connector service of the ACCUS ICP, which provides as output a RDF file with the information of the subsystem according to the nSSOO.

When the ACCUS ICP registers the information about the subsystem in the semantic subsystem and service repository, it automatically assigns and sends an identification number (ID) for the registered subsystem.

The information contained in *SRegistryRequest* will include information to identify the subsystem such as its geolocation, functionality, provider, owner, cost or security policies. This information can be sent in some machine-readable format such as XML or JSON.

#### 3.5.2. Services Registration

**Figure 12:**
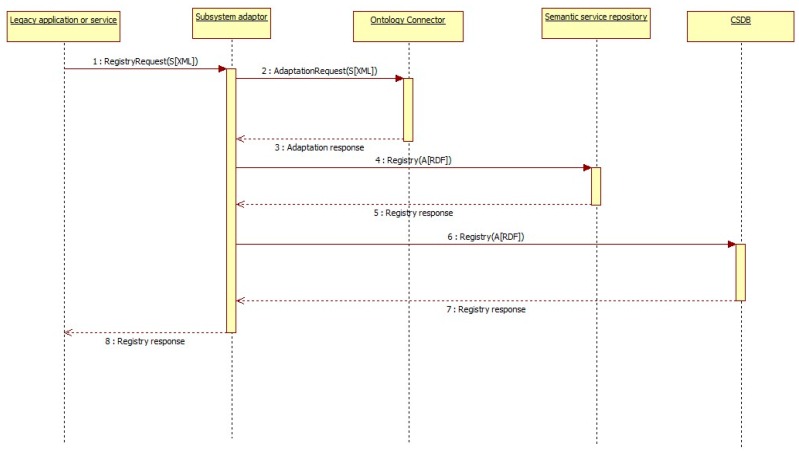
Service registration sequence diagram.

When a subsystem becomes registered in the ACCUS ICP, all legacy applications and services provided by the subsystem must be registered. As shown in Figure 12, first, each legacy application or service sends a registry request to the ACCUS ICP which includes the service profile in an XML document. Service profile contains information about the service or legacy application, such as service type, functionality or information about the operations that the service can do, along with the input and output parameters involved in each operation. This information is not compliant with the ACCUS ontology, so it must be adapted to the ACCUS ontology for the service registration to have it stored in the semantic service repository as well as in the CSDB.

Once the ACCUS ICP registers the information about the service or legacy application in the semantic subsystem and service repository, it will automatically assign and send an identification number (ID) for the registered service or legacy application, as well as for each one of the operations it provides.

The information contained in *RegistryRequest* will include information to identify the service or legacy application about its profile, business logic and context. As in subsystems registry, this information can be sent in some machine-readable format such as XML or JSON.

#### 3.5.3. Subsystem and Service Discovery

**Figure 13:**
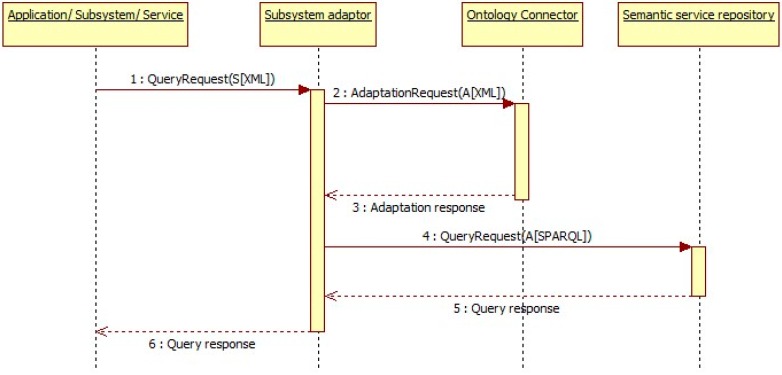
Discovery sequence diagram.

When a subsystem, service or application wants to know about other legacy applications, services or subsystems registered in ACCUS ICP, as shown in Figure 13, it will send a SPARQL query request towards the ACCUS ICP and the ACCUS ICP will send the query request towards the Semantic Subsystem and Service Repository. Semantic Subsystem and Service Repository in the ACCUS ICP will respond with the set of results of the query in XML format. These results depend on the query executed towards the Semantic Subsystem and Service Repository. There could be different types of queries depending on the information that subsystems or applications want to obtain, e.g., *list all registered subsystems, list all registered services, list all services of a specific subsystem, retrieve all the information about a specific subsystem* or *service* or just *retrieve some specific data*.

## 4. Example and Validation of Subsystem and Service Registration and Discovery

In this section, a complete example of a subsystem and a service registration and discovery is presented, based on the implementation done for the Semantic Subsystem and Service Repository of the ACCUS ICP. More specifically, using an example, the whole registration and discovery procedures will be shown, both for subsystems and services focusing on the input and output data formats, with the advantage of using specific data. Finally, validation done for evaluating the implementation will be also displayed.

### 4.1. Description of a Use Case Example

The proposed scenario involves a traffic control urban subsystem, which provides a traffic lights control service for managing the traffic lights cycles, along with information about the traffic density in several road intersections of a city. A smart mobility application uses the information provided by the traffic control subsystem in order to adapt traffic lights cycles to reduce and avoid traffic jams in the city.

Information provided by the traffic control subsystem is obtained by means of a Wireless Sensor Network, whose nodes will be deployed in road intersections of the city. The measurements taken by the sensors are sent via radio from the mesh network to the gateway, which comes with a special wireless node performing the base station role. This gateway is in charge of gathering data from the sensors and storing data until it is sent to the server and acts as an interface between ACCUS ICP and the Wireless Sensor Network. Then, the server, where all the data provided from the different sensors is collected and permanently stored, serves the data to the ACCUS ICP per request.

Both smart mobility application and traffic control subsystems are connected to the ACCUS ICP, which facilitates the communication between them with the services the platform provides. In order to enable this communication, traffic control subsystem must be registered in the ACCUS ICP first, so that it can be discovered and used by the smart mobility application.

Once the traffic control subsystem is connected, it sends a registration request, along with information about the subsystem, to the ACCUS ICP by means of its Subsystem Adaptor, using the corresponding method provided by the Application Interface. This method uses the Ontology Connector service to adapt the subsystem data sent in the request to an ACCUS ICP compliant format. Then, the Enterprise Service Bus locates and sends the request to the Semantic Subsystem and Service Repository, which registers the subsystem information received in a semantic repository, assigns an ID to the subsystem and returns it to the subsystem.

After subsystem registration, the traffic lights control service the subsystem provides is registered following the abovementioned procedure. When the service registration is completed, a smart mobility application can discover both the registered subsystem and service by querying Semantic Subsystem and Service Repository with SPARQL queries. In that case, information about the registered subsystem and service is returned to the smart mobility application in XML format.

**Figure 14:**
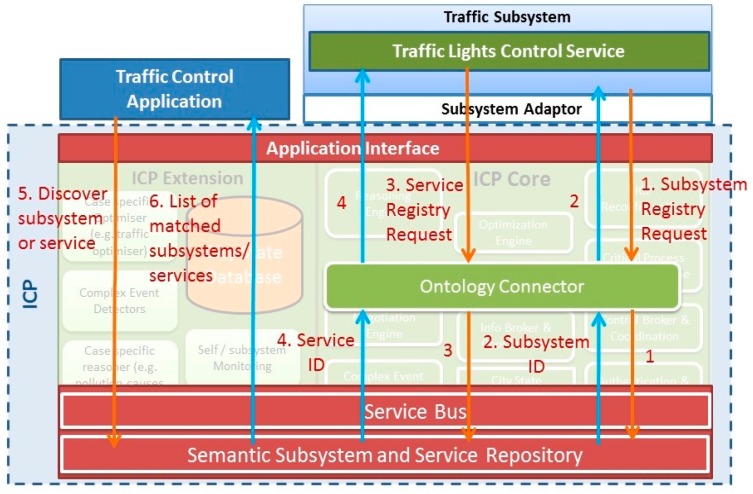
Example use case and registration and discovery processes.

Figure 14 shows the use case example, the whole registration and discovery processes as well as the interactions among the subsystem, the smart mobility application and the ACCUS ICP components involved in these processes.

### 4.2. Example of Subsystem and Service Registration and Discovery

**Figure 15:**
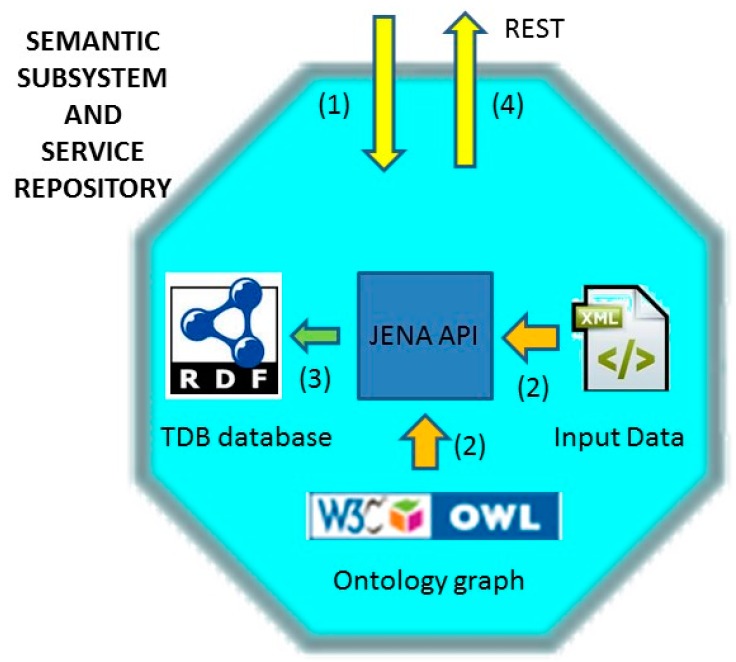
Semantic Subsystem and Service Repository Registration diagram.

Following the use case example presented in the previous section, and assuming that a) there is a traffic control subsystem that provides a traffic lights control service that b) has been just connected to the ACCUS ICP, two actions are carried out. As shown in Figure 15, first, the subsystem is discovered by the ICP and secondly, the subsystem sends a registration request to the ICP with information about it. Table 1 shows the information sent by the subsystem as well as the semantic annotations that the Semantic Subsystem and Service Repository will use to store that information.

Again, this information can be sent in some machine readable format as, for example, in XML or JSON. ACCUS ICP receives this data via a REST interface, and Semantic Subsystem and Service Repository converts the input data into semantically annotated data formatted in RDF. To do so, Jena API methods are used in order to obtain the classes defined in the ontology graph and stored in an .owl file, as well as to instantiate them with the values of the input data. Finally, the data is stored in a triple store database provided by Jena, called Jena TDB, and a unique ID is assigned to the subsystem and returned to it through the REST interface. This interface is also used whenever a subsystem, service or application connected to the ACCUS ICP wants to discover another subsystem, service or application registered in the ICP.

Once the traffic control subsystem has been registered, the traffic lights control service it provides is registered and, for that purpose, it sends a registration request to the ACCUS ICP with the information about it. Table 2 shows the information sent by the service as well as the semantic annotations that the Semantic Subsystem and Service Repository will use to store that information.

Then, when the ACCUS ICP receives the data, Semantic Subsystem and Service Repository registers the service, following the same procedure as for the subsystem, and finally an ID is assigned to the subsystem’s service.

As soon as the subsystem and the service it provides have been registered, they can be discovered by any subsystem, service or application connected to the ACCUS ICP, thus obtaining information about them. To do so, they can query the Semantic Subsystem and Service Repository, using one of the methods that this service API provides. The results will be output in a document XML.

For example, if the smart mobility application wants to discover the traffic control subsystem previously registered, it can call listAllSubsystems method, which returns a list of all registered subsystems, or also it can call subsystemInfo method, which returns the information about the subsystem whose ID matches the one passed as an input parameter.

Therefore, calling, for example, the latter method using the traffic control subsystem ID, the following SPARQL query is executed:

PREFIX ns: <http://www.semanticweb.org/ACCUS/1.1#>
SELECT ?subsystemID ?subsystemFunctionality ?subsystemHealthState ?subsystemGeolocation
?subsystemProvider ?subsystemOwner ?subsystemCost ?subsystemPolicies
WHERE {ns:@id@ ns:isSSIDOf ?subsystem.
?subsystem ns:hasSSID ?subsystemID.
?subsystem ns:hasSSHealthState ?subsystemHealthState.
?subsystem ns:hasSSContext ?subsystemContext.
?subsystemContext ns:hasSSGeoLocation ?subsystemGeolocation.
?subsystem ns:hasSSProfile ?subsystemProfile.
?subsystemProfile ns:hasSSFunctions ?subsystemFunctionality.
?subsystemProfile ns:hasSSCost ?subsystemCost.
?subsystemProfile ns:hasSSPolicies ?subsystemPolicies.
?subsystemProfile ns:hasSSOwner ?subsystemOwner.
?subsystemProfile ns:hasSSProvider ?subsystemProvider.}
		

And the following output will be returned:

<?xml version=*"1.0"*?>
    <sparql xmlns=*"http://www.w3.org/2005/sparql-results#"*>
      <head>
      <variable name=*"subsystemID"*/>
      <variable name=*"subsystemFunctionality"*/>
      <variable name=*"subsystemHealthState*"/>
      <variable name=*"subsystemGeolocation"*/>
      <variable name=*"subsystemProvider*"/>
      <variable name=*"subsystemOwner"*/>
      <variable name=*"subsystemCost"*/>
      <variable name=*"subsystemPolicies"*/>
      </head>
      <results>
      <result>
      <binding name=*"subsystemID"*>
      <uri>http://www.semanticweb.org/ACCUS/1.1#3309</uri>
      </binding>
      <binding name=*"subsystemFunctionality"*>
      <uri>
      http://www.semanticweb.org/ACCUS/1.1#Traffic Control Subsystem
      </uri>
      </binding>
      <binding name=*"subsystemHealthState"*>
      <uri>http://www.semanticweb.org/ACCUS/1.1#Active</uri>
      </binding>
      <binding name=*"subsystemGeolocation"*>
      <uri>
      http://www.semanticweb.org/ACCUS/1.1#Latitude: 54.3521 Longitude:
      18.64637
      </uri>
      </binding>
      <binding name=*"subsystemProvider"*>
      <uri>http://www.semanticweb.org/ACCUS/1.1#ACCUS</uri>
      </binding>
      <binding name=*"subsystemOwner"*>
      <uri>http://www.semanticweb.org/ACCUS/1.1#Company1</uri>
      </binding>
      <binding name=*"subsystemCost"*>
      <uri>http://www.semanticweb.org/ACCUS/1.1#Free</uri>
      </binding>
      <binding name=*"subsystemPolicies"*>
      <uri>http://www.semanticweb.org/ACCUS/1.1#Policy1, Policy2</uri>
      </binding>
      </result>
      </results>
    </sparql>
      

Similarly, if an application, subsystem or service wants to discover the traffic lights control service previously registered, it can call, the listAllServices method which returns a list of all registered services, or also the serviceInfo method, which returns information about the service whose ID matches the one passed as an input parameter. In the first case, the SPARQL query executed for listing all the registered services is the following:

PREFIX ns: <http://www.semanticweb.org/ACCUS/1.1#>
SELECT ?serviceID ?serviceFunctionality ?serviceType ?serviceHealthState ?serviceKind ?serviceCost ?securityProfile
WHERE {?Resource ns:hasSServiceType ?serviceType.
?Resource ns:hasSCost ?serviceCost.
?Resource ns:hasSHealthstate ?serviceHealthState.
?Resource ns:hasSProfile ?serviceProfile.
?serviceProfile ns:hasSID ?serviceID.
?serviceProfile ns:hasServiceFunctionality ?serviceFunctionality.
?serviceProfile ns:hasSKind ?serviceKind.
?serviceProfile ns:hasSecurityProfile ?securityProfile.}
		
and the information returned will be the following:

<?xml version=*"1.0"*?>
<sparql xmlns=*"http://www.w3.org/2005/sparql-results#"*>
 <head>
 <variable name=*"serviceID"*/>
 <variable name=*"serviceFunctionality"*/>
 <variable name=*"serviceType"*/>
 <variable name=*"serviceHealthState"*/>
 <variable name=*"serviceKind"*/>
 <variable name=*"serviceCost"*/>
 <variable name=*"securityProfile"*/>
 </head>
 <results>
 <result>
 <binding name=*"serviceID*">
 <uri>http://www.semanticweb.org/ACCUS/1.1#4713</uri>
 </binding>
 <binding name=*"serviceFunctionality"*>
 <uri>http://www.semanticweb.org/ACCUS/1.1#Subsystem and Service
 		Repository: an ontology translator for ACCUS ICP data treatment,
 		whenever the nSSOO ontology is required, that will be storing semantic information related with subsystems and services connected to the ICP.
 </uri>
 </binding>
 <binding name=*"serviceType*">
 <uri>http://www.semanticweb.org/ACCUS/1.1#ACCUS ICP service</uri>
 </binding>
 <binding name=*"serviceHealthState"*>
 <uri>http://www.semanticweb.org/ACCUS/1.1#Active</uri>
 </binding>
 <binding name=*"serviceKind"*>
 <uri>http://www.semanticweb.org/ACCUS/1.1#ACCUS compliant</uri>
 </binding>
 <binding name=*"serviceCost"*>
 <uri>http://www.semanticweb.org/ACCUS/1.1#Free</uri>
 </binding>
 <binding name=*"securityProfile"*>
 <uri>http://www.semanticweb.org/ACCUS/1.1#ACCUS security profile</uri>
 </binding>
 </result>
 <result>
 <binding name=*"serviceID"*>
 <uri>http://www.semanticweb.org/ACCUS/1.1#8647</uri>
 </binding>
 <binding name=*"serviceFunctionality"*>
 <uri>http://www.semanticweb.org/ACCUS/1.1#Traffic lights control </uri>
 </binding>
 <binding name=*"serviceType"*>
 <uri>http://www.semanticweb.org/ACCUS/1.1#Subsystem service</uri>
 </binding>
 <binding name=*"serviceHealthState*">
 <uri>http://www.semanticweb.org/ACCUS/1.1#Active</uri>
 </binding>
 <binding name=*"serviceKind"*>
 <uri>http://www.semanticweb.org/ACCUS/1.1#Not ACCUS compliant</uri>
 </binding>
 <binding name=*"serviceCost"*>
 <uri>http://www.semanticweb.org/ACCUS/1.1#Free</uri>
 </binding>
 <binding name=*"securityProfile"*>
 <uri>http://www.semanticweb.org/ACCUS/1.1#securityProfile1</uri>
 </binding>
 </result>
 </result>......</result>
 </result>......</result>
 </results>
</sparql>
		

Note that, when listing all services, information about Semantic Subsystem and Service Repository is also shown because internal ACCUS ICP active services are also registered in the semantic repository once the ICP is up.

### 4.3. Validation

In order to validate the practical performance of the Semantic Subsystem and Service Repository implementation, both the response time and the registration rate of the service have been tested. In order to test the response time, the timespan used for the different operations that the service provides to be executed has been measured. For each measurement, three samples have been taken in order to obtain an average value of the response time. On the other hand, the registration rate refers to the percentage of subsystems and services registered with regards to a certain number of registry requests done.

Tests have been done using an Intel(R) Core(TM)2 Quad CPU Q6600 processor @ 2.40 GHz equipped with 2.39 GHz and a RAM memory of 4 GB in a machine operating under the 64-bit Windows 7 Professional operating system.

#### 4.3.1. Response Time

When a request is done to the Semantic Subsystem and Service Repository for the first time, this service must be initialized, so it takes more time than usual to execute the request. So, to begin with, the response time required when a request is done to the semantic repository for the first time has been measured. Results obtained are shown in Table 3.

Now, considering that the service has been initialized, the response time of the semantic repository operations has been measured in three different cases: when the repository has 100, 500 and 1000 of subsystems and services registered. The purpose is to test the normal operation of the semantic repository for different amounts of registered data. The results obtained are shown in Table 4, Table 5 and Table 6.

Analyzing the results obtained, several conclusions can be drawn. Besides the fact that the response time is higher when the first request is done due to the initialization of the semantic repository, it can also be appreciated that the response time increases with the amount of subsystems and services stored.

Comparing the time response between the different operations, it can be seen that the time response of *listAll* operation is considerably higher than in the other operations due to the higher amount of data that must be retrieved, which are all the subsystems and services stored in the semantic repository, while, for example, in *listAllSubsystems* and *listAllServices* only operation subsystems in the first case, and services in the second case are shown. Regarding the last two operations mentioned, the time response of *listAllSubsystems* is higher than the time response of *listAllServices* because in the first operation more information is shown than in the second operation. For the same reason, time response in *registerService* and *getServiceInfo* operations is higher than in *registerSubsystem* and *getSubsystemInfo*. Finally, it can be appreciated that the time response of *registerSubsystem*, *registerService*, *getSubsystemInfo*, *getServiceInfo* does not change significantly with the amount of data registered, because in these operations just one subsystem or service is registered or queried so they are not affected by the amount of data registered.

#### 4.3.2. Registration Rate

For testing the registration rate, 5000 subsystem and service registry requests were done towards the Semantic Subsystem and Service Repository, and all the requests were successfully executed, registering the 100% of subsystems and services that requested registration.

## 5. Conclusions and Future Work

This paper has presented an ontology-based and automatic system for subsystem and service registry and discovery within the context of the ACCUS project. This proposed system, embedded in ACCUS ICP, is able to dynamically register and discover heterogeneous subsystems and services provided by subsystems within a Smart City so that cross-domain applications and collective optimization can be built upon the ICP by using existing services. To address the heterogeneity (e.g., data formats and protocols) of subsystems and services, a new ontology, named nSSOO, has been proposed and employed by the system to provide a formalized vocabulary for the registration and discovery processes. The nSSOO has been developed on the basis of three existing ontologies, including OWL-S, CityGML, and NRL. By complying with this ontology, heterogeneous subsystems and services provided by individual subsystems can share a same understanding which results in a formal and homogeneous appearance of the ICP. The proposed ontology, from a global point of view, is an important contribution to achieve semantic interoperability in the ICP and it has been presented with detailed explanations for inner composition. In addition to that, different software components which form the whole system have been shown with their main functionalities explained. The proposed ontology-based scheme for subsystem and service discovery and registry has been elaborated with a set of sequence diagrams that present the specific workflow of inner components involved in this scheme. Furthermore, after presenting the specific procedures to ease the subsystem and service discovery and registry, a complete example about discovering and registering a traffic control system and a traffic lights control service has been provided to show the performance of the proposed scheme. Different kinds of queries for information stored in ICP have also been introduced.

The system proposed to register and discover subsystems and services has been proven to be useful to interconnect different subsystems and services. What is more, it could abstract the heterogeneity of different subsystems and services so as to provide a homogeneous interface for applications or other services inside the ICP. Though this scheme aims to create an accurate reference framework for available information (e.g., subsystems, services, and applications) within the Smart Cities, it could be also possible to adapt it to other domains, such as underwater robotics. Developers can become aware of the services that are working in the Smart city, which are their features and how can be accessed, to design their applications by making use of those services provided by subsystems. Also, developers are isolated of the problem of transforming data protocols.

Future work could be focused on the following aspects:

- The proposed subsystem and service discovery and registry scheme should be tested in more scenarios in a real city. In ACCUS project, the city that has been chosen to deploy the pilot is Gdansk, in Poland.
- The relationships among different services are a crucial factor for application developers when they design brand new applications. Future work should focus on examining the similarity degree of different services. For instance, services able to provide similar functionalities can be alternatives if the ideal service to be used is not available. A potential solution could be including information about relationships of different services in relevant service profiles.
- The nSSOO ontology should evolve to richly describe more features of subsystems and services. For example, it is possible to extend nSSOO with some new classes using FOAF [38] to include additional aspects of information about people, such as roles like provider or owner. Another potential extension of the ontology could be including concepts about event-driven services.
- Future emphasis can also be put on including decision-making related algorithms in the nSSOO ontology. e.g., MADISE [39] ontology can be reused and integrated with the nSSOO ontology.

## Figures and Tables

**Table 1:** Subsystem data and semantic annotations.

Semantic Annotations	Subsystem Data
SS_Geolocation	Latitude: 54.3521 Longitude: 18.64637
SS_HealthState	Active
SubsystemFunctionality	Traffic Control Subsystem
SS_Provider	ACCUS
SS_Owner	Company1
SS_Cost	Free
SS_Policies	Policy1, Policy2

**Table 2:** Subsystem data and semantic annotations.

Semantic Annotations	Service Data	Semantic Annotations	Service Data
ServiceType	Subsystem service	OperationDescription	changeState: provides the change and duration of the new state
S_HealthState	Active	ParameterPrecondition	initialState
S_Cost	Free	ParameterInput	dataTimeInterval
ServiceKind	ACCUS compliant	ParameterOutput	lightValue
ServiceFunctionality	Traffic lights control	Static	Static
SecurityProfile	securityProfile1	Dynamic	Non dynamic
GroundingDescription	change state	IndoorLocation	Non indoor
GroundingInputMessage	none	OutdoorLocation	Outdoor
GroundingOutputMessage	changes done	ContextCriticality	Critical
GroundingURI	ACCUS/trafficLightsControl	Smartspace	SS2
GroundingProtocol	REST	S_Latitude	54.3521
SimpleProcess	Simple	S_Longitude	18.64637

**Table 3:** Response time when the first request is done to the semantic repository.

Operation	T1 (ms)	T2 (ms)	T3 (ms)	Average Time Elapsed (ms)
registerSubsystem	130	29	56	71.67
registerService	142	142	92	125.33
listAll	469	530	532	510.33
listAllSubsystems	450	447	463	453.33
listAllServices	476	490	460	475.33
getSubsystemInfo	464	498	476	479.33
getServiceInfo	815	578	564	652.33

**Table 4:** Response time when there are 100 subsystems and services registered.

Operation	T1 (ms)	T2 (ms)	T3 (ms)	Average Time Elapsed (ms)
registerSubsystem	84	30	31	48.33
registerService	47	53	46	48.67
listAll	284	105	138	175.67
listAllSubsystems	67	100	74	80.33
listAllServices	20	17	18	18.33
getSubsystemInfo	8	5	5	6
getServiceInfo	99	42	27	56

**Table 5:** Response time when there are 500 subsystems and services registered.

Operation	T1 (ms)	T2 (ms)	T3 (ms)	Average Time Elapsed (ms)
registerSubsystem	67	66	28	53.67
registerService	76	75	41	64
listAll	603	246	158	335.67
listAllSubsystems	84	70	76	76.67
listAllServices	73	63	62	66
getSubsystemInfo	6	3	5	4.67
getServiceInfo	183	44	26	84.33

**Table 6:** Response time when there are 1000 subsystems and services registered.

Operation	T1 (ms)	T2 (ms)	T3 (ms)	Average Time Elapsed (ms)
registerSubsystem	78	27	35	46.67
registerService	145	42	42	76.33
listAll	398	368	771	512.33
listAllSubsystems	195	500	367	354
listAllServices	138	107	111	118.67
getSubsystemInfo	6	4	5	5
getServiceInfo	108	18	16	47.33
